# A hub-attachment based method to detect functional modules from confidence-scored protein interactions and expression profiles

**DOI:** 10.1186/1471-2105-11-S1-S25

**Published:** 2010-01-18

**Authors:** Chia-Hao Chin, Shu-Hwa Chen, Chin-Wen Ho, Ming-Tat Ko, Chung-Yen Lin

**Affiliations:** 1 Institute of Information Science, Academia Sinica, No. 128 Yan-Chiu-Yuan Rd., Sec. 2, Taipei 115, Taiwan; 2 Division of Biostatistics and Bioinformatics, National Health Research Institutes. No. 35 Keyan Rd. Zhunan, Miaoli County 350, Taiwan; 3 Institute of Fishery Science, College of Life Science, National Taiwan University, No. 1, Roosevelt Rd. Sec 4, Taipei, Taiwan; 4 Department of Computer Science and Information Engineering, National Central University, No.300, Jung-da Rd, Chung-li, Tao-yuan 320, Taiwan; 5 Research Center of Information Technology Innovation, Academia Sinica, No. 128 Yan-Chiu-Yuan Rd., Sec. 2, Taipei 115, Taiwan

## Abstract

**Background:**

Many research results show that the biological systems are composed of functional modules. Members in the same module usually have common functions. This is useful information to understand how biological systems work. Therefore, detecting functional modules is an important research topic in the post-genome era. One of functional module detecting methods is to find dense regions in Protein-Protein Interaction (PPI) networks. Most of current methods neglect confidence-scores of interactions, and pay little attention on using gene expression data to improve their results.

**Results:**

In this paper, we propose a novel hub-attachment based method to detect functional modules from confidence-scored protein interactions and expression profiles, and we name it HUNTER. Our method not only can extract functional modules from a weighted PPI network, but also use gene expression data as optional input to increase the quality of outcomes. Using HUNTER on yeast data, we found it can discover more novel components related with RNA polymerase complex than those existed methods from yeast interactome. And these new components show the close relationship with polymerase after functional analysis on Gene Ontology.

**Conclusion:**

A C++ implementation of our prediction method, dataset and supplementary material are available at http://hub.iis.sinica.edu.tw/Hunter/. Our proposed HUNTER method has been applied on yeast data, and the empirical results show that our method can accurately identify functional modules. Such useful application derived from our algorithm can reconstruct the biological machinery, identify undiscovered components and decipher common sub-modules inside these complexes like RNA polymerases I, II, III.

## Background

In the post-genome era, there are many high-throughput data such as yeast two-hybrid, genetics interaction and gene expression microarray data are generated. Therefore, analysis of these data becomes an important research issues. One of major analyses is detecting functional modules on biological networks. Taking a protein as a vertex and connecting any two proteins that have direct interaction by an edge, we can build a Protein-Protein Interaction (PPI) network from a protein interaction dataset. Research evidence shows that biological systems are composed of functional modules [1,2]. Proteins in a module work together to perform certain biological functions. The interactions among these module components (proteins in this module) must be frequent. Based on this idea, a functional module should induce dense regions on the PPI network. Hence, detecting a densely connected cluster is a good heuristics to find protein functional module.

Algorithms in graph/network analysis have been applied in identifying essential functional modules from biological networks. For the divisive cluster method, it takes the whole network as a cluster at its beginning stage, and then split the cluster into smaller ones repeatedly until the network meet its stop criterion. Based on this idea, Dunn *et al*. [3] investigated biological function using Girvan and Newman's Edge-Betweenness algorithm which removes the edges with the highest edge-betweenness in each iteration. On the contrary, for the agglomerative clustering method, every single vertex forms a cluster at the beginning stage, and clusters are allowed to merge and grow as bigger as possible under certain constraints. The CPC (Clique Percolation Clustering method)[4,5], SCAN (Structural Clustering Algorithm for Networks) [6], COACH (COre-AttaCHment based method) [7], CMC (Clustering-based on Maximal Cliques)[8] and Core (Core-Attachment approach)[9] are classified into this category. There is a fusion strategy which combines the divisive and agglomerative approach, such as MoNet (Modular organization of protein interaction Networks) [10]. In the first stage of MoNet, it removes an edge with the highest edge-betweenness and pushes the edge into a stack until there is no edge can be removed. In the second stage, an edge is popped from stack and then adds into graph under certain condition.

Besides those methods mentioned above, there are many other functional module-detecting methods such as MCL (Markov CLuster algorithm) [11,12], MATISSE (Module Analysis via Topology of Interactions and Similarity SEts) [13], CEZANNE (Co-Expression Zone ANalysis using NEtworks)[14], and MST extension [15]. Based on a simulation of flow in graphs, MCL partitions the PPI network into many non-overlapping dense clusters. By finding proteins with highly similar gene expressions, MATISSE and CEZANNE generate non-overlapping clusters. According to maximum spanning trees calculated from weighted PPI networks, MST extension algorithm produces overlapping clusters. Recently, Gavin et al. [16] suggested that a protein complex consists of two parts, a core and an attachment. There are many researchers are based on this concept to design their own detecting protein complex algorithms, such as COACH (COre-AttaCHment based method) [7] and Core (Core-Attachment approach) [9]. These kinds of methods are also belonged to agglomerative method because a cluster grows from a core. This concept is also adopted in our algorithm.

Some previous studies showed that current PPI networks contain certain rate of false positive and false negative interactions [17,18]. However, most current functional module detecting methods from protein interactions pay little attention on this precondition. In addition, many clustering methods do not allow a vertex assigned to multiple clusters, but a protein may play roles in different ways. Therefore, functional modules may overlap with each other. To resolve these issues, we developed a novel agglomerative clustering method to detect functional modules from confidence-scored protein interactions. We conducted our approach on the PPI network came from Collins *et al*. [19] and gene expression data from MATISSE website [35]. The idea of Gavin et al. [16] on protein complex is also included in the algorithm. Our method can perform better than other existed ones to reconstruct the components and sub-complexes inside the protein complexes.

## Methods

### Preliminary assumptions of HUNTER algorithm

If the input data contains gene expression data, then we first remove PPI's edges if the two end vertices are expressed inconsistently, judged by the Pearson correlation threshold *t*. The target PPI is the cleaned PPI if the input data contains gene expression data; otherwise, the target PPI is the input data. We assume that a target PPI network *G *is a weighted graph with the vertex set *V*, the edge set *E*, and an edge weight function *w*. The neighbours of a vertex *v *are denoted by *N*(*v*). For a vertex set *S *⊆ *V*, *N*(*S*) denotes the vertex set (∪_*v *∈ *S*_*N*(*v*))-*S *and |*S*| denote its cardinality.

### Generating module seeds

Firstly, we want to generate a module seed *MS*(*v*) for each vertex *v *∈ *V*. Because the interactions among these module components are frequent, we assume that a protein functional module is connected in a PPI. Firstly, consider the gene expression complete graph consists of vertices having gene expressions, in which each edge is associated with the Pearson correlation of gene expressions of its end vertices as weight. For a vertex set *S *in the gene expression complete graph and a vertex *u *∈ *S*, the Bad Module Seed Index *BMSI*(*S*, *u*) is defined as the number of incident edges of *u *with weights less than or equal to a threshold *t*. For each connected component *NCC *of *N*(*v*), we keep removing vertex *u *∈ *NCC *with the maximum *BMSI*(*NCC*, *u*) from *NCC *until there is no vertex whose *BMSI *is bigger than zero. After the vertex removing process, we generate the resulted vertex set *N*'(*v*). Let Target Neighbor *TN*(*v*) denote the collection of connected component of *N'*(*v*). A vertex set *S *⊆ *V *is *q*-*connected *if the probability is at least *q *for all *U *⊂ *S *with at least one edge that connects *U *with *S *- *U *[14]. Let *MQC*(*v*) ⊆ *TN*(*v*) be a maximal *q*-connected. If |*MQC*(*v*)| is larger than 1, module seed *MS*(*v*) is the *MQC*(*v*) ∪ {*v}*; otherwise, *MS*(*v*) is an empty set.

### Criteria for module seeds growing and amalgamating

Next, we allow the module seed growing. The criteria for cluster expanding follow the idea proposed by Radicchi *et al *[20]. Briefly, for a vertex *v *∈ *V*, a vertex *u *can be joined to *MS*(*v*) if *u *is closed related to *MS*(*v*). Specifically speaking, we join a vertex *u *∈ *N*(*MS*(*v*)) into *MS*(*v*) if 2 × |*N*(*u*) ∩ *MS*(*v*)| > |*MS*(*v*)|. In an iteration, all vertices satisfying the criteria are joined to *MS*(*v*) at one time. The grown *MS*(*v*) is used as *MS*(*v*) in the next iteration. We continue this process until no more vertex can be joined into *MS*(*v*). A module seed *MS(v) *⊂ *V *is a weak community if . The module seeds *MS*(*v*) qualified as weak communities are left as grown modules.

Grown modules may overlap on some vertices. For any two grown module *U*_*i *_and *U*_*j*_, we merge them into a larger grown module if 2 × |*U*_*i *_∩ *U*_*j*_| > min {|*U*_*i*_|, |*U*_*j*_|}. We go through the process until there are no grown modules can be merged. The collection of resulted modules, the final modules, forms the clustering of our module detection method.

### Enrichment on gene ontology terms

Gene ontology (GO) project aims on standardizing the annotation of genes across species and databases based on an expert-curated mechanism [21]. Using a set of controlled vocabulary, attributes of a gene product are described in three different aspects, the elemental, biochemical activities of a gene product at the molecular level (Molecular Function, MF), the biological processes that a gene or gene product contributes (Biological Process, BP), and the location where the gene product can be found (Cellular Component, CC) in different depth. These terms are arranged hierarchically, like directed acyclic graphs (DAG) in which the vertex may have multiple parents and multiple relationships to their parents. In addition, each term inherits all the relationships of its parent(s). In this study, we use the retrieval of GO terms of a clustering, the relatedness of GO terms in clusters, and the enrichment of terms in a cluster to evaluate the performance of module detecting methods in GO::TermFinder [22]. The GO ontology file (gene_ontology.obo) and annotation file (gene_ontology.sgd) used in this study are updated version released on 07/29/2009 and 07/25/2009, respectively.

### F-measure

For an ontology *d*, we denote the total number of proteins whose annotation in the ontology *d *by *N*. Given a term *a*, we denote the total number of proteins whose annotations contain this term as *M*. Given a cluster *b*, we denote the number of proteins in the cluster as *n *and the number of proteins whose annotations contain term *a *as *x*. The *p*-value, defined in equation (1), is the probability of observing *x *or more proteins in the cluster *b*, given the ontology *d *and term *a *by random [22]:(1)

Sensitivity is defined as the fraction of annotations that are enriched in at least one cluster at *p*-value < 10^-4^, and specificity is defined as the fraction of clusters enriched with at least one annotation at *p*-value < 10^-4 ^[14]. Here we use F-measure, a weighted average of the sensitivity and specificity defined in equation (2), to evaluate the performance of GO term retrieval by functional modules:(2)

### Co-annotation

For a term *a *in an GO ontology category DAG, the probability *p*(*a*) is defined as the number of proteins associated with the term divided by the number of proteins associated with any term in the category DAG [23]. In order to make a comparison on the relationship among terms, we scored the similarity between terms based on the equation proposed by Schlicker *et al *[23]. For a set of common ancestors of terms *a*_1 _and *a*_2 _is denoted as *S*(*a*_1_, *a*_2_), the similarity between two terms *a*_1 _and *a*_2 _is:(3)

The annotation score of a cluster is the average relevance similarity of all protein pairs in the cluster. The annotation score for a clustering is the weighted mean over all cluster annotation scores on a GO ontology. The co-annotation score for a clustering is the geometric mean of the clustering annotation scores on "biological process" and "molecular function" [24].

### Co-localization

If proteins in the same functional module work together, they should have high chance to show up at the same physical location [25]. We denote a localization data as *O *= {*O*_*k*_|*O*_*k *_is a set of proteins occur in location *k*} and a clustering generated by a detecting method as *C *= {*C*_*k *_|*C*_*k *_is a set of proteins classified in a predicted cluster}. We define the co-localization score of clustering *C *as follows:(4)

For a cluster *C*_*j*_,  is the maximum number of proteins in the cluster which are found at the same localization.

### Program source code and test datasets used in this study

The source code of HUNTER (Supplementary S1), dataset of protein interaction (Supplementary S2), gene expression (Supplementary S3) and other information are available in HUNTER website. Two extra datasets, MIPS [26] and Aloy *et al*. [27] are applied for validating module discovery methods. Protein complexes defined in these two datasets are used as the gold-standard protein complex sets.

## Results and Discussions

### Thresholds in HUNTER

There are two thresholds used in HUNTER. One is the *q*-connected threshold *q *used for finding module seeds, the other is the correlation threshold *t *used for filtering PPI's interactions. We set *q *as 0.95 corresponds to an "error probability" of 0.05. For any two proteins having interaction, we compute the Pearson correlation of gene expression if the expression data is available. The correlation threshold *t *is determined by the following method which is modified from Elo *et al *[28]. Suppose there are *r *gene expressions. First, we build a complete graph of *r *nodes, *K*_*r*_, in which each node represents a protein (and its expression) and each edge is associated with the Pearson correlation of expressions of its two end vertices. Let graph *H *be the sub-graph of *K*_*r *_with edges of Pearson correlation greater than a candidate correlation threshold *d*. We define a function *C*(*K*_*r*_, *d*) as follows (equation 5),(5)

where *v *is a vertex, deg(*v*) is the number of neighbours of vertex *v *and *E*_*v *_is the number of edges between the protein *v*'s neighbours in the graph *H*. In other word, *C*(*H*) is the clustering coefficient of the graph *H*. A graph *H*_0 _is a random graph which preserves the degree distribution of graph *H*, and a function *C*_0_(*K*_*r*_, *d*) = *C*(*H*_0_)[29]. The correlation threshold *t *in HUNTER is decided by the following formula:(6)

In a general speaking, the range of candidate correlation threshold *d *is from 0.6 to 0.99 [28]. In order to increase the speed of computing the correlation threshold *t*, we set *d*_*j *_= 0.6 + 0.01 × *j*, where *j *∈ [0, 39].

### Identification of functional modules

HUNTER method is designed for extracting functional modules from a weighted or unweighted PPI network with option for using gene expression data to increase the quality of outcomes. There are many methods for detecting functional modules. However, most of them work only on unweighted PPI networks, and few of them use gene expression data to help them to get better results. CEZANNE [14] is a recently published methodology that finds functional modules based on detecting co-expressed gene sets on a confidence-based interaction network. To make the result comparable, we use the same datasets that Ulitsky and Shamir [14] used for evaluating the performance of CEZANNE. The PPI network came from Collins *et al*. [19] and the gene expression data was downloaded from MATISSE website [35]. Briefly, the yeast PPI data contains 3625 proteins and 26149 interactions. The maximal connected component of this PPI network, composed of 3382 proteins with 26003 interactions, is used as the input set. There are 1300 proteins in this input set were found to have gene expression data.

**Figure 1:**
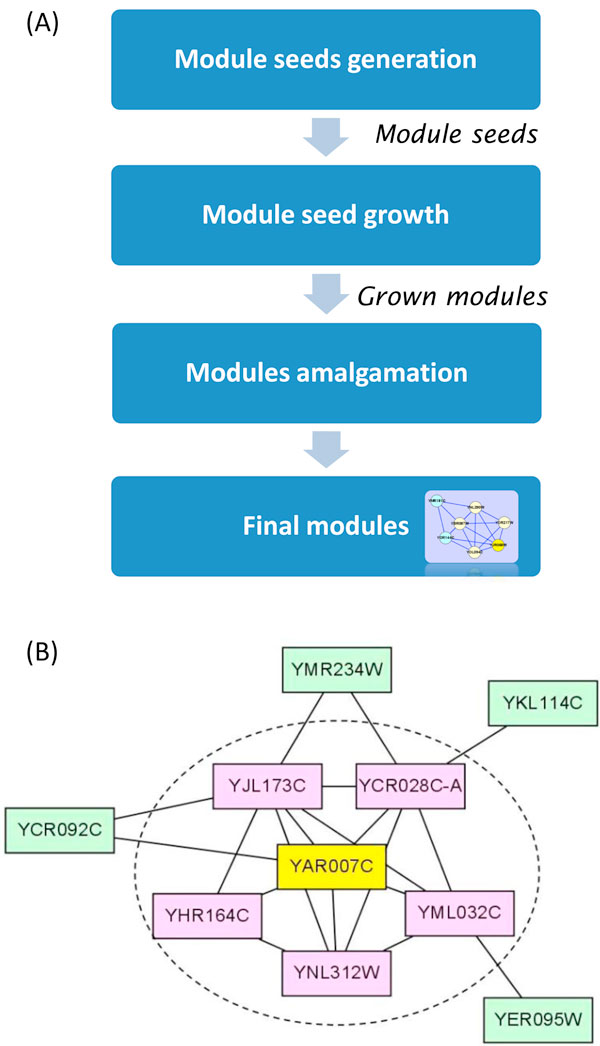
**A brief of HUNTER**. (A) The flowchart of HUNTER. (B) An example of DNA Replication Protein A (RPA), which is a highly conserved single-stranded DNA binding protein complex involved in DNA replication, repair, and recombination. An example of HUNTER predict cluster, a ten-protein cluster. The module seed of this cluster consists of one protein in yellow (YAR007C) and five proteins in pink (YJL173C, YCR028C-A, YML032C, YNL312W, and YHR164C) in the dashed circle. Among these six proteins, five proteins (YAR007C, YJL173C, YCR028C-A, YML032C, and YNL312W) form a fully connected subgraph (clique) in the PPI network. Proteins in green, YCR092C, YMR234W, YKL114C, and YER095W are the attachments to the module seed.

HUNTER method is divided into three main stages as shown in Figure 1. In the first stage, we generate a module seed for each vertex. Next, each module seed is allowed to grow by adding vertices with edges connected to the module seed if they show strong connection to the module. In other words, the outside connection of a grown module is less than the inside connection. In the last stage, we merge any two grown modules if they have many common vertices until no grown module can be merged. HUNTER found 52 functional module clusters, composed by 792 proteins, from the input yeast network (Supplementary S2). The modules are listed in Supplementary S4, in which 23 modules are matched to known complexes listed in MIPS database. An example of HUNTER-defined cluster (Cluster_15, Supplementary S4) is illustrated in Figure 1B. Ten components are clustered in this module. We found this module is involved in DNA replication, repair, and recombination. The core of the complex is one of the module seed in the initial stage. This module looks like a highly connected clique (dashed circle) with four attachments.

### Validation of hub-attachment structures

A hub protein is essential for cell viability. In our previous work, we demonstrated the size of a Maximum Neighbourhood Component (MNC) of a vertex *v *is positive correlated with the contribution of the vertex to individual in terms of viability [29]. Based on the concepts of MNC and *q*-connected, we create the definition of module seed. In this study, we propose that a module seed can be a "heart" of a cluster. Let a set *S *= {*S*_*i *_|*S*_*i *_⊂ *V *be a collection of subset of *V*, and the average similarity of *S *is defined as follows,(7)

The average similarity of interactions in PPI data, final modules and module seeds are calculated respectively. As shown in Table 1, the average similarity of a set composed of final modules is larger than the average similarity of the whole vertex set *V *as one component on both Biological Process and Cellular Component ontology. That means the relationships of proteins in a final module are statistically closer than the relationships of two random chosen proteins in a PPI network. The table 1 also shows that the average similarity of a set composed of module seeds is larger than the average similarity of a set composed of final modules. It means that a module seed is the "heart" of a cluster because the similarity of two proteins in the same module seed is very high in most cases. As the module illustrated in Figure 1B, using this method, we can generate hub-attachment structures by attaching all closer neighbours to the module seed they surrounded.

**Table 1:** Average similarity of interactions involved in PPI data (supplementary S2), final modules and module seeds. *V*: whole vertex, *S*: collection of subset of *V*.

*Set S*	*Biological Process*	*Cellular Component*
*S*_1 _= {{*V*}}	0.428	0.386
*S*_2 _= {*s*_*i*_|*s*_*i *_is a final module}	0.613	0.568
*S*_3 _= {*s*_*i*_|*s*_*i *_is a module seed}	0.692	0.697

### Evaluation on the performance of module discovery

Gene ontology term is a well-designed set of vocabulary to describe roles, functions and cellular locations of genes and gene products [21]. Proteins in a functional module are supposed to work together to perform some biological functions [30]. Therefore, the goodness of a functional module can be revealed by co-existence and the consistence of annotations among the components of a module.

**Figure 2:**
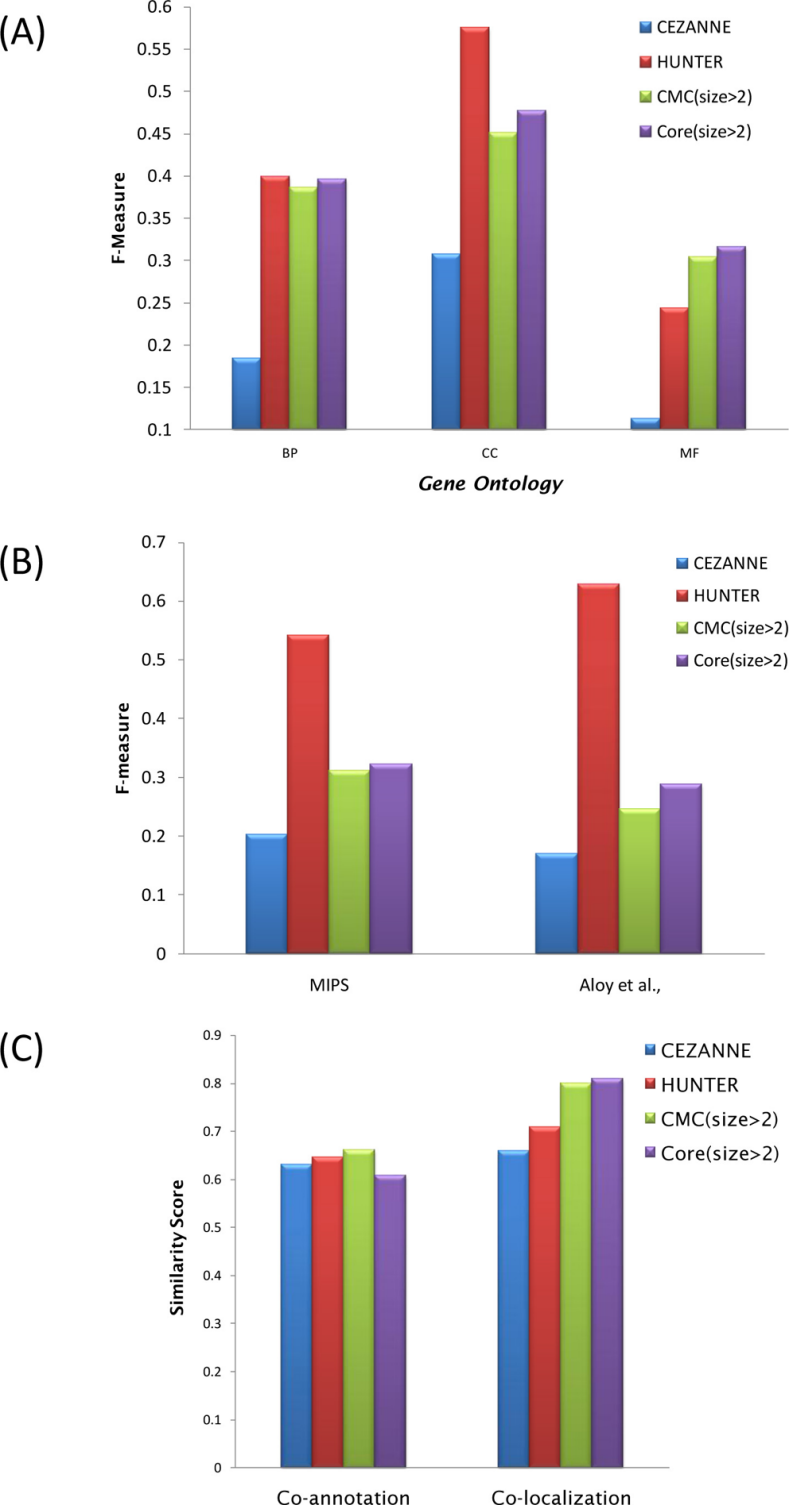
**The performance amid HUNTER and other methods**. (A) F-Measure with GO on test data. (B) F-Measure on Experimental Datasets. (C) The similarity scores of co-annotation and co-localization for each clustering by GO terms.

In this study, we use the retrieval of GO terms of a clustering, the relatedness of GO terms in a cluster, and the enrichment of terms in a cluster to evaluate the performance of module detecting methods. Firstly, we examine the accuracy, the recovery of meaningful GO terms, of the four module detecting methods, HUNTER, CEZANNE, CMC and Core, using F-measure. Terms in three GO categories are evaluated separately. As shown in Figure 2A and Supplementary S12, HUNTER got high scores in all three GO categories, and is the first ranked method of biological process and cellular component. Then, we take datasets from MIPS and Aloy *et al*. and the protein complex lists from these two sets are served as validated gold-standard sets. The performance of HUNTER is superior over the three others (Figure 2B). Next, we examine the similarity on annotations of proteins in a functional module. The similarity scores of GO terms for each clustering are calculated. As shown in Figure 2C, HUNTER also performs well. Furthermore, we check enrichment of GO terms for each module (Supplementary S5-S7). The highly enriched GO terms are arranged closely in the ontology, and most functional modules highlight one or few branches in ontology.

Table 2 shows the brief summary of the clustering of the four methods. We found that the complex number, proteins included in the complex, and the number of unique proteins is much lower in the results of CEZANNE and HUNTER. Methods of CMC and Core tend to produce lots of small, highly overlapped modules. In another word, methods like CMC and Core covered more module components (high recall rate) on the resulting clustering. The expansion on module lists leads to low precision rate while high F-measures are achieved. In contrast, CEZANNE method recovers less proteins from the PPI, but the modules discovered by CEZANNE are more possible to be true modules (precision rate > 0.5) than those from CMC and Core (precision rate at about 0.2 or less). We successfully enhance both the coverage of module components and precision on module discovery of HUNTER method (Supplementary S12, S13). Among the four methods, HUNTER is top ranked by F-measure in both datasets.

**Table 2:** The number of protein complexes, the total protein counts in complexes, and unique proteins in complexes in the gold-standard protein complex data and predicted protein complexes.

	*Number of Complex*	*Number of Protein*	*Number of Unique Protein*
***Protein Complexes***			
**Aloy *et al.***	78	626	588
**MIPS**	199	3165	1200
			
***Predicted Protein Complexes***			
**CEZANNE**	14	471	471
**HUNTER**	52	908	842
**CMC(size > 2)**	530	4145	1826
**Core(size > 2)**	434	2826	1964

The input datasets of this experiment are derived from Aloy *et al*. and MIPS, and are available in supplementary S10 and S11. Protein complex lists defined in these two datasets are used as gold-standard lists. The predictions based on the test interactome (supplementary S2) and the expression profile (supplementary S3) are available in supplementary S13 for the two dataset respectively.

### Analysis on RNA polymerase complexes

**Figure 3:**
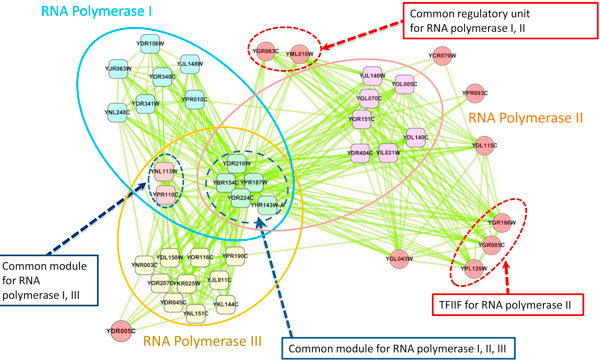
**Predicted RNA polymerase complex by HUNTER**. Major components in each complex can be distinguished in colors: Polymerase complexes I core (pink), Polymerase complexes II core (yellow), polymerases complex III core (blue), common sub-network for polymerase I, II and III (green), share components for I and III (red). Rectangles indicate the actual polymerase components validated by structural data, circles mean protein not previously reported and identified by HUNTER. Ellipses in blue, red and yellow indicate RNA polymerase I, II and III, respectively. Most components marked as red circle recognized by HUNTER were related with polymerase annotated by functional enrichment.

Gene transcription in eukaryotic cells is carried out by the three different DNA-dependent RNA polymerases Pol I, Pol II, and Pol III. These RNA polymerases are the central multi-protein machines that synthesize ribosomal, messenger, and transfer RNA, respectively [31]. Here, HUNTER identified a cluster (Cluster_35, Supplementary S4) of 41 proteins from the experiment protein network [19] and expression dataset [13], which effectively encloses the three RNA polymerase complexes (Figure 3). The components of each polymerases described the structural data [31] are marked by ellipses in blue (RNA Pol I), red (RNA Pol II) and yellow (RNA Pol III). All the 31 protein components mentioned in the structural data, including common sub-networks (5 proteins for all three polymerases, 2 for polymerase I and III), are found in the HUNTER resulting module (Cluster_35). One component, YDR005C, is described as a component of RNA Pol III in MIPS database. New components identified in HUNTER are marked as red spot with the protein IDs. We identified a common regulatory unit consisted of YGR063C - YML010W. The unit enriched GO categories shows that this unit mediates in both activation and inhibition of transcription elongation, plays a role in pre-mRNA processing, and stabilizes the polymerases. Three proteins grouped by red dashed circle in Figure 3, YGR186W, YGR005C and YPL129W, show a close binding relationships as TFIIF for RNA polymerase II on their annotation info. YDL115C (IWR1) interacts with many components in RNA Pol II and TFIIF complex; a similar conclusion has been reported in a previous TAP experiment [32]. YGL043 shows the high connectivity with RNA Pol II. According to GO annotation, it plays important roles on regulation of translation. Three HUNTER-identified components, YCR079W, YPR093C, and YDL115C, do not have GO annotation related to RNA synthesis process. However, the high connectivity of these vertexes to Pol II suggests the possibility of functional involvement in the regulation of enzyme activity.

Our result match to more polymerase components with fewer vertexes of low biological relevance in terms of experimental evidence and annotations in comparing with network study on DNA-directed RNA complex prediction by the extended MST approach [19]. Therefore, HUNTER modules may provide more insights for future research as what we expected. Figure 2. an induce graph from GO Molecular Function ontology.

## Conclusion

HUNTER method is designed for extracting functional modules from a weighted PPI network with option for using gene expression data to increase the quality of outcomes. The workflow of the algorithm implementation is described in Supplementary materials (S9). As mentioned in introduction, a protein network data is a collection of various sources of protein-protein interaction dataset derived from *in vivo*, *in vitro*, *in silico *(data mining), and etc. Noises from false-positive/false-negative and regardless of the dynamic nature of gene expression are obvious error sources on the inferred network. Two noise-reducing strategies are adopted in our method. First, HUNTER accepts a protein interaction network with the interaction probability. The probability of protein interaction may derived by statistical or modelling strategies such as domain-domain interaction and interlogous inferring methods. The probability model makes help in the network feature detection. Besides, the consistence in the expression pattern of genes provides hints of gene co-existence. Previous reports showed that proteins in the same complex have similar gene expression patterns [33,34]. HUNTER started with network feature detecting procedure with the reference from expression data to define the starting module seeds. That will help module detecting method to a reasonable baseline of co-existence of module components.

Modules detected by HUNTER are hub-attached, that means the modules contains proteins of cardinal importance in the protein network. The performance of HUNTER is superior to CEZANNE in GO annotation retrieval and the average of relatedness of GO terms within a module. We have further examine the functional modules detected by HUNTER; half of the modules are found to be known protein complex in interaction database MIPS. As mentioned in previous section, HUNTER successfully identified a module that covers all known components of three RNA polymerase complexes. Several components in this module are highly related to the polymerase core, which may act as candidates for regulators on enzyme activity.

In summary, HUNTER can identify functional modules accurately. It is flexible in the input network of either weighted or unweighted interaction dataset, with or without gene expression dataset. It is a useful tool for researchers to expand their research target into a functional structure of an interactome and will help to find new components involved in a protein complex.

## Competing interests

The authors declare that they have no competing interests.

## Authors' contributions

CHC and CYL conceptualized the algorithm, design the method, drafted the manuscript together with SHC. CHC was responsible for the implementation. CWH and MTK participated in discussion and conceptualization as well as revising the draft. All the authors read and approved the manuscript.

## References

[B1] Barabasi A-L Oltvai ZN Network biology: understanding the cell's functional organization Nat Rev Genet 2004 11 2 101 113 10.1038/nrg1272 14735121

[B2] Rives AW Galitski T Modular organization of cellular networks Proceedings of the National Academy of Sciences of the United States of America 2003 11 3 1128 1133 10.1073/pnas.0237338100 12538875 PMC298738

[B3] Dunn R Dudbridge F Sanderson CM The use of edge-betweenness clustering to investigate biological function in protein interaction networks BMC Bioinformatics 2005 11 39 10.1186/1471-2105-6-39 15740614 PMC555937

[B4] Palla G Derenyi I Farkas I Vicsek T Uncovering the overlapping community structure of complex networks in nature and society Nature 2005 11 7043 814 818 10.1038/nature03607 15944704

[B5] Zhang S Ning X Zhang X-S Identification of functional modules in a PPI network by clique percolation clustering Computational Biology and Chemistry 2006 11 6 445 451 10.1016/j.compbiolchem.2006.10.001 17098476

[B6] Mete M Tang F Xu X Yuruk N A structural approach for finding functional modules from large biological networks BMC Bioinformatics 2008 11 Suppl 9 S19 10.1186/1471-2105-9-S9-S19 18793464 PMC2537570

[B7] Wu M Li X Kwoh CK Ng SK A core-attachment based method to detect protein complexes in PPI networks BMC Bioinformatics 2009 11 169 10.1186/1471-2105-10-169 19486541 PMC2701950

[B8] Liu G Wong L Chua HN Complex discovery from weighted PPI networks Bioinformatics 2009 11 15 1891 1897 10.1093/bioinformatics/btp311 19435747

[B9] Leung HC Xiang Q Yiu SM Chin FY Predicting protein complexes from PPI data: a core-attachment approach J Comput Biol 2009 11 2 133 144 10.1089/cmb.2008.01TT 19193141

[B10] Luo F Yang Y Chen CF Chang R Zhou J Scheuermann RH Modular organization of protein interaction networks Bioinformatics 2007 11 2 207 214 10.1093/bioinformatics/btl562 17092991

[B11] Dongen S Graph Clustering by Flow Simulation 2000 University of Utrecht, The Netherlands

[B12] Enright AJ Van Dongen S Ouzounis CA An efficient algorithm for large-scale detection of protein families Nucl Acids Res 2002 11 7 1575 1584 10.1093/nar/30.7.1575 11917018 PMC101833

[B13] Ulitsky I Shamir R Identification of functional modules using network topology and high-throughput data BMC Syst Biol 2007 11 8 10.1186/1752-0509-1-8 17408515 PMC1839897

[B14] Ulitsky I Shamir R Identifying functional modules using expression profiles and confidence-scored protein interactions Bioinformatics 2009 11 9 1158 1164 10.1093/bioinformatics/btp118 19297352

[B15] Friedel CC Zimmer R Identifying the topology of protein complexes from affinity purification assays Bioinformatics 2009 11 16 2140 2146 10.1093/bioinformatics/btp353 19505940 PMC2723003

[B16] Gavin A-C Aloy P Grandi P Krause R Boesche M Marzioch M Rau C Jensen LJ Bastuck S Dumpelfeld B Proteome survey reveals modularity of the yeast cell machinery Nature 2006 11 7084 631 636 10.1038/nature04532 16429126

[B17] Suthram S Shlomi T Ruppin E Sharan R Ideker T A direct comparison of protein interaction confidence assignment schemes BMC Bioinformatics 2006 11 360 10.1186/1471-2105-7-360 16872496 PMC1550431

[B18] von Mering C Krause R Snel B Cornell M Oliver SG Fields S Bork P Comparative assessment of large-scale data sets of protein-protein interactions Nature 2002 11 6887 399 403 10.1038/nature750 12000970

[B19] Collins SR Kemmeren P Zhao XC Greenblatt JF Spencer F Holstege FC Weissman JS Krogan NJ Toward a comprehensive atlas of the physical interactome of Saccharomyces cerevisiae Mol Cell Proteomics 2007 11 3 439 450 17200106 10.1074/mcp.M600381-MCP200

[B20] Radicchi F Castellano C Cecconi F Loreto V Parisi D Defining and identifying communities in networks Proc Natl Acad Sci USA 2004 11 9 2658 2663 10.1073/pnas.0400054101 14981240 PMC365677

[B21] Ashburner M Ball CA Blake JA Botstein D Butler H Cherry JM Davis AP Dolinski K Dwight SS Eppig JT Gene ontology: tool for the unification of biology. The Gene Ontology Consortium Nat Genet 2000 11 1 25 29 10.1038/75556 10802651 PMC3037419

[B22] Boyle EI Weng S Gollub J Jin H Botstein D Cherry JM Sherlock G GO::TermFinder--open source software for accessing Gene Ontology information and finding significantly enriched Gene Ontology terms associated with a list of genes Bioinformatics 2004 11 18 3710 3715 10.1093/bioinformatics/bth456 15297299 PMC3037731

[B23] Schlicker A Domingues FS Rahnenfuhrer J Lengauer T A new measure for functional similarity of gene products based on Gene Ontology BMC Bioinformatics 2006 11 302 10.1186/1471-2105-7-302 16776819 PMC1559652

[B24] Friedel CC Krumsiek J Zimmer R Bootstrapping the interactome: unsupervised identification of protein complexes in yeast J Comput Biol 2009 11 8 971 987 10.1089/cmb.2009.0023 19630542

[B25] Kumar A Cheung KH Ross-Macdonald P Coelho PS Miller P Snyder M TRIPLES: a database of gene function in Saccharomyces cerevisiae Nucleic Acids Res 2000 11 1 81 84 10.1093/nar/28.1.81 10592187 PMC102388

[B26] Mewes HW Amid C Arnold R Frishman D Guldener U Mannhaupt G Munsterkotter M Pagel P Strack N Stumpflen V MIPS: analysis and annotation of proteins from whole genomes Nucleic Acids Res 2004 32 Database D41 44 10.1093/nar/gkh092 14681354 PMC308826

[B27] Aloy P Bottcher B Ceulemans H Leutwein C Mellwig C Fischer S Gavin AC Bork P Superti-Furga G Serrano L Structure-based assembly of protein complexes in yeast Science 2004 11 5666 2026 2029 10.1126/science.1092645 15044803

[B28] Elo LL Jarvenpaa H Oresic M Lahesmaa R Aittokallio T Systematic construction of gene coexpression networks with applications to human T helper cell differentiation process Bioinformatics 2007 11 16 2096 2103 10.1093/bioinformatics/btm309 17553854

[B29] Lin CY Chin CH Wu HH Chen SH Ho CW Ko MT Hubba: hub objects analyzer--a framework of interactome hubs identification for network biology Nucleic Acids Res 2008 36 Web Server W438 443 10.1093/nar/gkn257 18503085 PMC2447731

[B30] Hartwell LH Hopfield JJ Leibler S Murray AW From molecular to modular cell biology Nature 1999 11 6761 Suppl C47 52 10.1038/35011540 10591225

[B31] Cramer P Armache KJ Baumli S Benkert S Brueckner F Buchen C Damsma GE Dengl S Geiger SR Jasiak AJ Structure of eukaryotic RNA polymerases Annu Rev Biophys 2008 11 337 352 10.1146/annurev.biophys.37.032807.130008 18573085

[B32] Krogan NJ Cagney G Yu H Zhong G Guo X Ignatchenko A Li J Pu S Datta N Tikuisis AP Global landscape of protein complexes in the yeast Saccharomyces cerevisiae Nature 2006 11 7084 637 643 10.1038/nature04670 16554755

[B33] Jansen R Greenbaum D Gerstein M Relating whole-genome expression data with protein-protein interactions Genome Res 2002 11 1 37 46 10.1101/gr.205602 11779829 PMC155252

[B34] Tornow S Mewes HW Functional modules by relating protein interaction networks and gene expression Nucleic Acids Res 2003 11 21 6283 6289 10.1093/nar/gkg838 14576317 PMC275479

[B35] MATISSE website http://acgt.cs.tau.ac.il/matisse/

